# Associations between adverse childhood experiences and progression to incident psychiatric disorders in older adults: A 22-year cohort study

**DOI:** 10.21203/rs.3.rs-7307635/v1

**Published:** 2025-09-02

**Authors:** Hui-Ying Fan, Mu-Rui Zheng, Xiao-Xuan Meng, Qinge Zhang, Zhi-Cheng Du, Zhaohui Su, Teris Cheung, Gabor Ungvari, Chee H. Ng, Yu-Tao Xiang

**Affiliations:** University of Macau; Beijing anding hospital; Southeast University; University Notre Dame Australia; University of Melbourne; Unit of Psychiatry, Faculty of Health Sciences

**Keywords:** adverse childhood experiences, psychiatric disorder, older adults, disease progression, comorbidities

## Abstract

The long-term impact of adverse childhood experiences (ACEs) on the development of psychiatric disorders in older adults remains unclear. This study examined associations between ACEs and incident psychiatric disorders in older adults (PDOA) over 22 years. Data from the 2000–2022 Health and Retirement Study (HRS) were analyzed. Time-varying Cox regression and multistate Markov models were applied to explore the impact of ACEs on transitions across five health states: healthy, physical conditions (PC), mental symptoms (MS), comorbid PC & MS, and PDOA. Models were adjusted for demographic, behavioral, and disease-related factors. Among 8,628 participants during average 16.8-year follow-up, 1,429 developed psychiatric disorders (incidence: 9.85 per 1,000 person-years). ACEs, particularly trauma/violence (aHR = 1.279) and family dysfunction (aHR = 1.358), were significantly associated with higher risk. A dose-response relationship was found. Participants with ACEs had higher transition percentages and intensities from healthy to less healthy states, notably from PC & MS to PDOA (percentages: 3.7% vs. 3.2%) and from the healthy state to MS (intensities: 0.130 vs. 0.104). They also spent less time in the healthy state and more time in comorbid states, with a 33% higher 22-year cumulative probability of PDOA (25.3% vs. 19.0%). Risk was further elevated by younger age, female sex, higher educational level, low physical activity, insomnia, lung diseases, and arthritis. ACEs appear to have enduring adverse impacts on mental health in later life by accelerating the progression to comorbidity and the development of psychiatric disorders. Early screening and physicalmental health interventions are essential for prevention.

## 1. Introduction

With the rapidly aging population worldwide, poor mental health in older adults has become a major public health challenge, contributing substantially to high disability, low quality of life and increased healthcare utilization^1^. Previous research found that approximately 14% of individuals aged 60 and above suffer from psychiatric disorders globally^2^. The 2019 Global Health Estimates indicated that mental health conditions, particularly depression and anxiety, accounted for 10.6% of the total disability-adjusted life years (DALYs) among older adults^1^. Mental health in later life is largely influenced by physical conditions, social environments as well as the cumulative effects of life experiences. Factors, such as exposure to adversity, loss of intrinsic capacity and declining functional ability, can lead to increased psychological distress^1^. Among the early life factors, adverse childhood experiences (ACEs) have emerged as a critical determinant of mental well-being with lasting impact on mental health across the lifespan.

ACEs refer to experiences of potentially traumatic events occurring before age 18 years, which can disrupt a person’s sense of safety, stability, and bonding^3^. Common types of ACEs include child abuse, neglect, or household dysfunction involving substance abuse, mental illness, incarceration, domestic violence and parental separation or divorce^3, 4^. Additionally, research on ACEs has expanded the scope to other significant stressors such as exposure to natural disasters, extreme economic hardship, bullying, school and community violence, life-threatening illnesses or injuries, and even school grade retention^5, 6^. Exposure to ACEs can increase the risk of a wide range of psychiatric problems, including depression, anxiety, post-traumatic stress disorder and substance use disorders^7–12^. For example, a large birth cohort study found that each additional ACE was associated with a 52% increase in the odds of developing any psychiatric disorder in adulthood, even after adjusting for shared genetic and environmental influences^12^. Neuroimaging research has shown that ACEs can lead to alterations of blood flow, activity, and connectivity in specific brain regions which may potentially mediate the impact of ACEs on the development of psychiatric disorders^13, 14^.

Despite the growing body of evidence, most studies have focused on the associations between ACEs and mental health issues among children, adolescents, young and middle-aged adults, with relatively few longitudinal studies tracking the progression of psychiatric disorders into later life^15^. In addition, most studies used cross-sectional designs or short follow-up periods, which limit the ability to capture the dynamic changes in mental health status over time. There is a lack of research on how ACEs can influence the transition between different mental states in older populations, such as from subclinical symptoms to a diagnosed psychiatric disorder. Furthermore, late-life psychiatric disorders often co-occur with chronic physical illnesses, which may affect the progression and management of psychiatric disorders^16^.

To address these gaps, we conducted a cohort study using follow-up data from the nationally representative Health and Retirement Study (HRS) across a 22-years period to: (1) examine the associations between different ACEs and the incidence of psychiatric disorders, incorporating time-varying covariates and repeated assessments; and (2) explore how ACEs influence the changes across various health states (e.g., physical and mental health symptoms transitioning to psychiatric disorders). Findings from this study could inform the development of preventive measures to mitigate the adverse impact of ACEs, and intervention strategies to improve the mental health outcomes in older populations.

## 2. Methods

### 2.1 Study design and samples

This was a cohort study based on the most recent 22-year dataset (2000–2022; waves 5–16) of the Health and Retirement Study (HRS). The HRS is a nationally representative longitudinal study that has biennially surveyed U.S. residents aged 50 and older since 1992 (Wave 1). The study is funded by the National Institute on Aging (grant numbers NIA UO1AG009740 and NIA RO1AG073289) and is conducted by the University of Michigan^17^. The details of the HRS methodology have been described previously (Heeringa and Connor, 1995). Our study used the data obtained from the RAND HRS Longitudinal File (Version P, including 2000–2020 wave of sociodemographic and health-related data), HRS core data (including ACEs-related data and 2022 wave of all related data) and exit data (including all-cause mortality data). The RAND HRS Longitudinal File, developed by the RAND Center for the Study of Aging, provides systematically cleaned and processed data from all waves of the HRS except for the 2022 wave^18^.

The study cohort included individuals who participated in the 2000 wave (i.e., baseline) and followed up longitudinally to 2022. Participants who fulfilled the inclusion criteria: 1) were aged 50 years or older at baseline; 2) were interviewed during at least one follow-up assessment; 3) completed ACEs assessments during the 2008–2012 surveys; and 4) had no psychiatric disorders at baseline. For covariates with missing values related to demographic, health, or lifestyle variables, we applied imputation using last observation carried forward (LOCF) and next observation carried forward (NOCF), as previously recommended^19, 20^. The HRS received ethical approval from the Institutional Review Board of Virginia Commonwealth University (HM20023839). All participants provided informed consent. As a secondary analysis of HRS data, this study was exempt from ethical review.

### 2.2 Measurements

#### 2.2.1 Adverse childhood experiences

Following previous studies^21, 22^, the presence of ACEs was assessed using nine items extracted from the Leave-Behind Questionnaires (LBQ)^23, 24^ that was completed during the 2008–2012 waves of the HRS. Participants were asked whether they had ever experienced the events before 18 years old or that the year of ACE occurrence before 18 years old was recorded in the HRS database. All items were handled as binary variables (1=“Yes” and 0= “No”). The total scores of ACEs ranged from 0 to 9, with higher scores indicating more severe ACEs. As per previous research^6, 24, 25^, ACEs were classified into three types: Trauma & violence (4 items including “been in a major fire or disaster”, “fire a weapon in combat”, “ever victim of physical attack”, and “life-threatening illness”), Family dysfunction (3 items including “mother neglect”, “parents drink or use drugs and caused family problems”, and “physical abused by parents”) and Social problem (2 items including a year of school over again, and trouble with the police). The description and coding of the ACE items are listed in Table S1.

#### 2.2.2 Incidence of psychiatric disorders

Following previous research^26^, psychiatric disorders were identified based on the standardized question at each HRS wave: “Have you been diagnosed with emotional, nervous, or psychiatric problem by a doctor?”. The study outcome was the incidence of psychiatric disorders which was first identified during follow-up.

#### 2.2.3 Covariates

##### 2.2.3.1 Sociodemographic data

Sociodemographic variables potentially related to psychiatric disorders included age, sex (male vs. female), race (White/Caucasian, Black/African American or other), marital status (categorized as married, divorced/separated, widowed, or never married/other), educational level (> 12 years vs. ≤12 years), living with others (yes vs. no), vigorous physical activity (≥ 3 times/week, yes vs. no), alcohol use (yes vs. no) and cigarette smoking (yes vs. no).

##### 2.2.3.2 Health-related variables

Chronic diseases included self-reported physician-diagnosed major physical diseases recorded in the HRS such as diabetes, cancer, heart disease, stroke, hypertension, lung diseases, and arthritis.

Depression was evaluated using the 8-item Center for Epidemiologic Studies Depression Scale (CESD-8)^27^. Each item was scored as 1 or 0 (yes or no), with items 4 and 6 being reverse scored. The total scores ranged from 0 to 8, with higher scores indicating more severe depressive symptoms. A cutoff score of 4 was used to identify individuals having depressive symptoms (depression hereafter)^28^.

Cognitive function was assessed using the modified version of the Telephone Interview for Cognitive Status (TICS-m)^29^. Following a previous study^30^, the total score of TICS-m was calculated by adding up the respective scores of memory, attention and calculation domains, which ranged from 0 to 27, with higher scores indicating better cognitive performance. A cutoff score of 11 was used to identify individuals having cognitive impairment^31, 32^.

Sleep was measured using the 4-item Jenkins Sleep Scale (JSS-4)^33^. Each item was rated on a 3-point Likert scale ranging from 1 (“most of the time”) to 3 (“rarely or never”). Item 4 was reverse scored. Higher total scores reflected more severe insomnia symptoms, and a cutoff score of 5 was used to identify those with insomnia symptoms (insomnia hereafter)^34^. All covariates were assessed at each HRS biennial wave.

### 2.3 Statistical Analyses

Frequencies and percentages as well as means and standard deviations (SDs) were used to describe categorical and continuous variables, respectively. All covariates were compared between different ACE types using Chi-square, *t* and Wilcoxon rank sum tests, as appropriate. All statistical analyses were conducted using R (version 4.4.2)^35^. A two-sided *P*-value < 0.05 was considered statistically significant.

To examine the longitudinal association between ACEs and the risk of psychiatric disorders in older age (PDOA), we used Cox proportional hazards regression models with time-varying covariates^36^. Participants were followed up from baseline (2000) until the occurrence of a psychiatric disorder, death, loss to follow-up, or end of study (2022), whichever came first. Time-varying covariates, such as health-related variables and lifestyle factors, were updated at each wave to account for the changes over time. Three Cox models were constructed, adding covariates progressively. Model 1 adjusted for age, sex and race; Model 2 added marital status, education level, living with others, lifestyle variables (i.e., smoking, alcohol use, and vigorous physical activity) based on Model 1; Model 3 further added major physical diseases (i.e., diabetes, cancer, heart conditions, stroke, hypertension, lung diseases, and arthritis), depression (i.e., total CESD-8 score), insomnia (i.e., total JSS-4 score) and cognitive function (i.e., total TICS-m scores) based on Model 2. Following previous studies^12, 37^, ACEs were analyzed in three forms: (1) ACE type (i.e., no ACEs, Trauma & violence, Family Dysfunction, Social problems) with no ACEs group as the reference; (2) total number of ACEs (i.e., 0, 1, 2, and 3 + ACEs) as a categorical variable with the 0 ACE group as the reference, and (3) continuous ACE scores (ranging, 0–9). Schoenfeld residuals were used to assess the proportional hazards assumption and to ensure no violation was found. Kaplan-Meier survival curves were plotted to examine potential differences in incidence rates across types and number of ACEs.

We also conducted subgroup analyses to explore whether the associations between ACEs and the incidence of PDOA were moderated by age (50–64 years vs. ≥65 years), sex, marital status, educational level, living with others, alcohol use, cigarette smoking, engagement in vigorous activity, insomnia (JSS-4 scores < 4 vs. ≥4), depression (CESD < 5 vs. ≥5), and cognitive impairment (TICS-m ≤ 11) between the group with and without ACEs.

To assess the robustness of the associations between ACEs and the incidence of psychiatric disorders, five sensitivity analyses were conducted by: (1) excluding cases with psychiatric disorders during the first two years after the baseline; (2) excluding cases with psychiatric disorder in the first follow-up wave; (3) using the total number of physical diseases as a time-varying covariate in Model 3, instead of adjusting for individual physical diseases; (4) using traditional Cox regression model adjusting only for baseline covariates; (5) performing competing risk analyses using Fine–Gray subdistribution hazard models, given that death may preclude the occurrence of psychiatric disorders. Cumulative incidence function (CIF) curves were constructed to compare the risks of psychiatric disorders and death overtime across different types and number of ACEs.

Additionally, the multistate Markov model was implemented to capture the dynamic transitions of health status among individuals with ACEs and those without ACEs, focusing particularly on how ACEs affected transitions across physical and mental health states and ultimately led to psychiatric disorders. Such an approach provides an integrated perspective on disease process and enables identification of influential risk factors and long-term predictions^38^.

In this study, each participant was followed up with repeated health assessments across multiple follow-up waves, and transitions between defined states were tracked accordingly. Five states were defined to represent key clinical trajectories: (1) absence of physical conditions or mental health symptoms (No PC & No MS); (2) presence of physical conditions only (PC); (3) presence of mental health symptoms only [MS, depression (CESD ≤ 4) and (or) cognitive impairment (TICS-m ≤ 11)]; (4) co-occurrence of both physical conditions and mental health symptoms (PC & MS); and (5) having psychiatric disorders, defined as the absorbing state. The state structure is shown in Fig. 2A. States 1 to 4 were defined as transient states, while state 5 as the absorbing state. Transition intensity parameters (q_*ij*_) represent the instantaneous transition risk from state *i* to *j*. These were estimated through maximum likelihood under the assumption of continuous time Markov processes. For instance, q_3→5_ reflects the instantaneous hazard of progressing from only having mental health symptoms to a diagnosed psychiatric disorder. Figure 2B illustrates representative individual-level transitions in our sample, showing the heterogeneous patterns of disease progression observed during follow-up. The transition intensity matrix *Q* based on 5-state model was formulated as follows:

$$Q=\left(\begin{array}{ccccc} -\left(q_{1\to 2}+q_{1\to 3}+q_{1\to 4}+q_{1\to 5}\right) & q_{1\to 2} & q_{1\to 3} & q_{1\to 4} & q_{1\to 5}\\ q_{2\to 1} & -\left(q_{2\to 1}+q_{2\to 3}+q_{2\to 4}+q_{2\to 5}\right) & q_{2\to 3} & q_{2\to 4} & q_{2\to 5}\\ q_{3\to 1} & q_{3\to 2} & -\left(q_{3\to 1}+q_{3\to 2}+q_{3\to 4}+q_{3\to 5}\right) & q_{3\to 4} & q_{3\to 5}\\ q_{4\to 1} & q_{4\to 2} & q_{4\to 3} & -\left(q_{4\to 1}+q_{4\to 2}+q_{4\to 3}+q_{4-5}\right) & q_{4\to 5}\\ 0 & 0 & 0 & 0 & 0 \end{array}\right)$$

In this context, the transition probability represents the likelihood of a transition from one state to another within a given time interval. To assess the influence of potential factors on transition dynamics, a series of univariate Markov models was fitted first, each incorporating a single covariate (e.g., ACEs in the total sample, and all other covariates in the ACE-exposed group) independently. Cumulative transition probabilities over a 22-year horizon were then computed. All multistate models were implemented using the R package *msm*.

## 3. Results

### 3.1 Participant characteristics

Of the 19,580 participants in the 2000 wave, we included 8,628 participants who followed-up with 79,477 observations from 2000 to 2022 (Figure S1). Baseline sociodemographic and health-related characteristics of participants stratified by the four ACE types are summarized in Table 1. The mean age of participants was 64.75 years (SD = 8.01), and the majority were female (n = 4,998; 57.2%), married (n = 6,310; 73.1%), and had lower than high school education (n = 4,901; 56.8%). Univariate analyses showed significant differences between ACE types in terms of age, sex, race, marital status, educational level, living status, vigorous physical activity, suffering from heart conditions, depression score and cognition score (*P* < 0.05).

### 3.2 ACEs and the incidence of psychiatric disorders in older adults

#### 3.2.1 Incidence rate of psychiatric disorders in older adults

During an average follow-up duration of 16.8 years, 1,429 (16.6%) participants experienced psychiatric disorders. The overall incidence rate was 9.85 (95%CI: 9.34–10.37) per 1,000 person-years. The incidence rate of PDOA increased with the total number of ACEs: 8.85 per 1,000 person-years for participants with no ACE, 11.2, 13.0 and 15.0 per 1,000 person-years for those with one, two, and ≥ 3 ACEs, respectively (Table S3). The incidence rate was also different between different ACE types: 11.4, 14.1, 8.9 per 1,000 person-years for those with trauma & violence, family dysfunction, and social problems, respectively (Table 2).

#### 3.2.2 The associations between ACEs and the incidence of psychiatric disorders in older adults

Table 2 shows significant associations between trauma & violence, and family dysfunction with the incidence of PDOA. In the fully adjusted model, participants with childhood trauma & violence, and family dysfunction had 27.9% and 35.8% higher risks of PDOA, respectively, as compared to those with no ACE exposure [adjusted Hazard ratio (aHR): 1.279, 95%CI: 1.052–1.555 vs. aHR: 1.358, 95%CI: 1.190–1.548]. However, no association was observed between childhood social problems and the incidence of PDOA. Table S2 shows a dose-response relationship between the total number of ACE and the incidence of PDOA, which persisted across all three models. In the fully adjusted model, participants with two and ≥ 3 ACEs had 27.9% and 50.6% higher risks of PDOA, respectively, compared to those with no ACE [aHR: 1.279, 95%CI: 1.056–1.549 vs. aHR: 1.506, 95%CI: 1.128–2.011]. The Kaplan-Meier survival curve (Figure S1) illustrates significant differences in incidence rates across different types and number of ACEs (*P* < 0.001).

**Figure 1:**
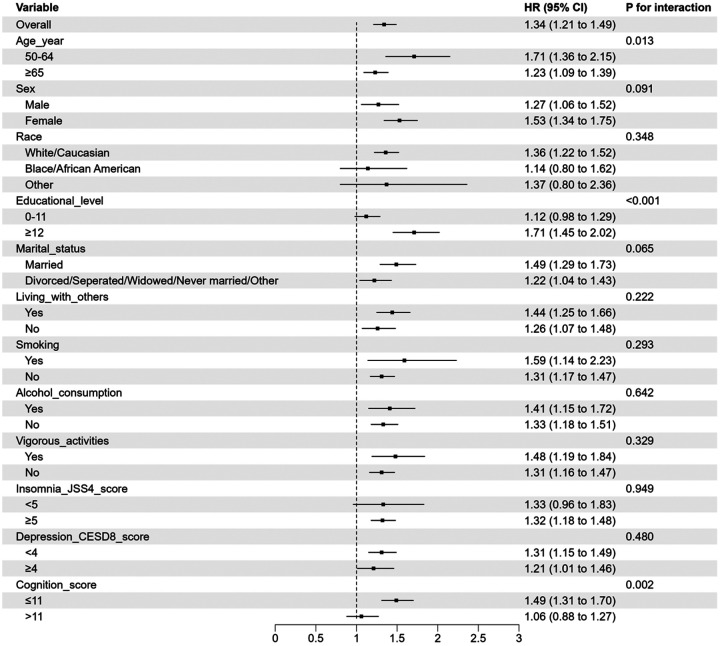
The association between ACEs and the incidence of PDOA stratified by demographic and clinical correlates Notes: Graphs show hazard ratios (HRs) and 95% confidence intervals (CIs) for psychiatric disorders in older adults. ACEs was assessed using a binary variable (yes vs. no). ACEs: adverse childhood experiences; PDOA: psychiatric disorders in older adults; HR: Hazard ratio; CI: Confidence interval; CESD-8: 8-item Center for Epidemiological Studies Depression Scale; JSS-4: 4-item Jenkins Sleep Scale.

**Figure 2:**
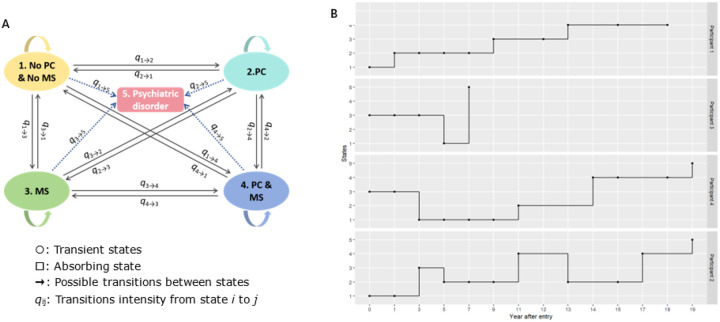
(A) Transitions among multi states in the Markov model: State 1 (No PC & No MS): participants without physical conditions (PC, including diabetes, stroke, heart conditions and cancers) and mental symptoms (MS, including depressive symptoms and cognitive impairment); State 2 (PC): participants with physical conditions; State 3 (MS): participants with mental symptoms; State 4 (PC & MS): participants with mental symptoms and physical conditions; State 5 (absorbing state): participants with psychiatric disorder. The arrows illustrate the possible transitions between the transient status and the absorbing state. Each transition parameter *q* illustrates the transition intensity and *q*_i→j_ is defined as the instantaneous transition risk from state i to j. (B) Possible transition paths for four selected subjects with ACEs. Observations were truncated once the absorbing states appeared. The nodes represent the actual observed states, with solid lines indicating potential transition trajectories observed during the follow-up period. Possible transition paths were selected randomly based on the recorded states. ACEs: adverse childhood experiences. Transitions between transient states and absorbing state (psychiatric disorder) (A) and possible transition paths for the selected subjects with ACEs (B)

Figure 1 shows that the association between ACEs and the incidence of psychiatric disorders was more pronounced among individuals aged 50–64 years (aHR: 1.71, 95% CI:1.36–2.15) than those aged ≥ 65 years (aHR: 1.23, 95% CI: 1.09 to 1.39; *P* for interaction: 0.013); among participants with higher educational level (aHR: 1.71, 95% CI: 1.45–2.02) than those with lower educational level (aHR: 1.12, 95% CI: 0.98–1.29, *P* for interaction: <0.001); and among those with cognitive decline (HR: 1.49, 95% CI: 1.31–1.70) compared to those without cognitive decline (aHR:1.06, 95% CI:0.88–1.27; *P* for interaction: 0.002).

In sensitive analyses, consistent associations between ACEs and the incidence of psychiatric disorders were observed (Tables S3-S6). The competing risk analyses showed that the association between ACEs and the incidence of PDOA remained significant when accounting for death as a competing event (Table S7). The CIF curves revealed a higher cumulative risk of PDOA over time in individuals with greater exposure to ACEs and those with family dysfunction or direct trauma & violence, although death became more prominent as a competing risk after approximately 100 months of follow-up (Figure S3).

### 3.3 Transitions between health states among participants with and without ACEs

#### 3.3.1 State transition percentage and intensity

The observed state transitions during follow-up are summarized in Table S8. Among participants with ACEs, transitions from comorbid physical conditions and mental health symptoms (PC & MS) to psychiatric disorders accounted for the largest proportion (3.7%), followed by transitions from MS alone (3.5%). In contrast, among those without ACEs, the proportion transitioning to psychiatric disorders was lower across all states, with the highest transition from the PC & MS state (3.2%). Participants with ACEs were also more likely to transition between vulnerable health states (e.g., from MS to PC & MS: 8.2%) compared to those without ACEs (7.5%). Participants with ACEs showed consistently higher transition intensities across most state transitions compared to those without ACEs, e.g., the transitions from No PC & No MS to MS (0.130 vs. 0.104) and from PC to PC & MS (0.146 vs. 0.129) (Table S9).

#### 3.3.2 State transition probability and total duration

**Figure 3:**
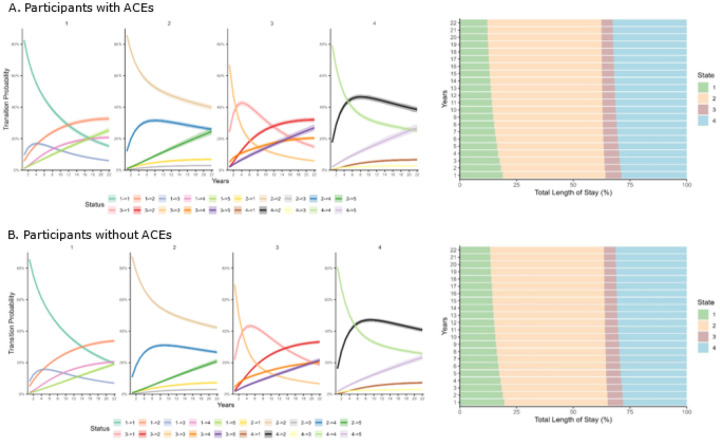
Transition probability curves and estimated percentage of total duration for five health states over 22 years Notes: State 1–5: 1. Participants without physical conditions (diabetes, stroke, heart conditions and cancers) and mental symptoms (depressive symptoms and cognitive impairment); 2. Participants with physical conditions; 3. Participants with mental symptoms; 4. Participants with mental symptoms and physical conditions; 5. Participants suffering from psychiatric disorder. ACEs: adverse childhood experiences.

Figures 3 (left panel) and S4 illustrate the 22-year transition probability curves across five health states, stratified by participants with ACEs and those without ACEs. The probability of remaining in the healthy state (1→1, No PC & No MS) declined sharply for both groups, especially in ACE group. At baseline, the probabilities of state 1 were over 0.8 in both groups, while by the year 2022, the probabilities dropped to 0.153 in the ACE group and 0.196 in the no-ACE group, respectively. The probability of transitioning from the healthy state to more adverse states (e.g., 1→2, 1→4, and especially 1→5) increased over time. Certain transitions, such as recovery to healthy states (e.g., 2→1, 3→1), remained low and stable in both groups, indicating more likelihood of accelerated health deterioration and less likelihood of recovery.

Notably, the group with ACEs exhibited higher and faster probabilities of progressing from the healthy state or any intermediate states to the absorbing state 5 (psychiatric disorders) over the 22 years; for instance, at 22 years, 1→5: ACE 0.253 vs. no ACE 0.190; 2→5: ACE 0.245 vs. no ACE 0.209; 3→5: ACE 0.269 vs. no ACE 0.215; 4→5: ACE 0.269 vs. no ACE 0.233. We calculated the 22-year cumulative transition probabilities from four transient health states (States 1–4) to the incidence of psychiatric disorders (State 5). The cumulative transition probabilities ranged from 19.0–23.3% and from 25.3–26.9% across states in the group without ACE and the group with ACEs, respectively. Even among participants initially in a healthy state (State 1), those with ACE exposure had a 33% higher risk of transitioning to psychiatric disorders (25.3% vs. 19.0%) by the year 2022. Furthermore, the group with ACEs exhibited consistent higher probabilities of transitioning between intermediate states (e.g., 1→2, 1→4, 3→4, and 2→4), indicating a higher likelihood of progression to early physical conditions, as well as comorbid physical and mental health conditions.

Figure 3 (right panel) shows the standardized percentages of total duration across the five states. Over the 22-year follow-up period, most of the duration was in state 2 (PC), accounting for approximately 48% of the total time across both groups. This was followed by state 4 (PC & MS), comprising around 31%, and state 1 (No PC & No MS), at about 15%. Hence, both total duration of PC and PC & MS prolonged, while those of healthy state (No PC & No MS) shortened. When comparing participants with ACEs and those without ACEs, the group with ACEs spent less time in the healthy state (state 1) (14.5% vs 15.6%) and more time in the comorbid state (state 4) (31.2% vs 30.1%) than their non-ACE counterparts. These trends indicated that ACEs might be associated with earlier onset and longer duration of complex comorbidity prior to the onset of psychiatric disorder.

#### 3.3.3 Covariate effects on state transitions

Across the whole sample, univariate Markov models demonstrated that individuals with ACEs had a significantly higher risk of developing both physical and mental health conditions. Specifically, participants with ACEs had 19.0%, 25.3% and 85.9% higher risks of transition from No PC & No MS to PC, MS, and to psychiatric disorder, respectively; and 13.8% higher risk of transition from PC to PC & MS (Table S10).

Among participants with ACEs, univariate models (Table S11) showed that older age (≥ 65 years) and male sex were consistently associated with a lower risk of transitioning from all states to psychiatric disorders (e.g., No PC & No MS → Psychiatric disorders, HR:0.538, 95% CI: 0.362–0.798 for age; HR:0.536, 95% CI: 0.359–0.802 for male). Vigorous physical activity was protective for developing psychiatric disorders among those with No PC & No MS (HR:0.620, 95% CI: 0.424–0.906). Insomnia (JSS-4 total score ≥ 5) significantly increased the risk of transition from combined physical and mental symptoms to psychiatric disorders (HR: 2.721, 95% CI: 1.244–5.952). Among physical diseases, lung diseases (HR: 2.592, 95% CI: 1.524–4.356) and arthritis (HR: 1.530, 95% CI: 1.028–2.275) were associated with a higher risk of psychiatric disorders from No PC & No MS state. High school education or above also increased the risk of transition from mental symptoms to psychiatric disorders (HR: 1.864, 95% CI: 1.114–3.120).

## 4. Discussion

To the best of our knowledge, this was the first cohort study to investigate the associations between ACEs and the incidence of psychiatric disorders in older adults, based on 22 years of follow-up data. We found that ACEs were strongly associated with an increased risk of psychiatric disorders in later life after adjusting for the time-varying confounders regarding lifestyle, physical and mental health conditions. We also identified effect modifications in the relationship between ACEs and the incidence of psychiatric disorders based on stratification by sociodemographic, lifestyle, and health-related factors. Moreover, we explored how ACEs influenced the transition from healthy and intermediate health states to psychiatric disorders and identified risk factors for such transitions.

Our study demonstrated that ACEs could significantly increase the risk of PDOA over a 22-year follow-up. Using time-varying Cox regression, we found that the total ACE score, cumulative number of ACEs and specific ACE types, such as trauma & violence and family dysfunction, were all significantly associated with an elevated risk of psychiatric disorders. The risk increased in a dose–response manner, with individuals exposed to three or more ACEs having the highest risk. The competing risk model further validated that these associations were not confounded by the differential risk of mortality across groups. These results are consistent with previous findings that ACEs contribute to poorer cognitive, physical, and mental health outcomes in later life^39^. In terms of ACE subtypes, childhood family dysfunction and trauma & violence were associated with the highest risks of psychiatric disorder. Due to the severe and chronic nature of ACEs, such adversities could cause profound disruptions in emotional regulation, early attachment, and neurobiological development, resulting in lasting impact on mental health in middle and older adulthood^40, 41^. In contrast, social problems (e.g., school difficulties, trouble with police) showed weaker or nonsignificant associations, probably because they are more transient or likely mediated by other factors such as social support or resilience^39^.

Our multistate Markov model revealed several key findings about the impact of ACEs on mental health trajectories in older adults. First, ACEs accelerated transitions toward PDOA, since older adults with ACEs transitioned more frequently and more rapidly from healthy to less healthy states. For example, transitions from comorbid physical and mental health conditions (PC & MS) to psychiatric disorders were more frequent in the group with ACEs (3.7% vs. 3.2%); transitions from the healthy state (No PC & No MS) to mental health symptoms (MS) were also faster in the group with ACEs (intensity: 0.130 vs. 0.104), underscoring the role ACEs in accelerating progression towards psychiatric disorders. Such results is aligned with previous research showing that ACEs accelerated the progression of both physical and mental health conditions, predisposing individuals to complex multimorbidity and psychiatric illness^39^.

Second, ACEs prolonged vulnerability states and reduced time in healthy state. The group with ACEs spent less time in the healthy state (14.5% vs. 15.6%) and more time in the comorbid PC & MS state (31.2% vs. 30.1%) over the study period. This pattern indicated that ACEs might not only influence the onset of psychiatric disorders but also prolong the periods of subclinical vulnerability, which overall likely exacerbate psychiatric risks.

Third, individuals with ACEs showed consistently higher cumulative risk of transitioning from healthy or intermediate states to having psychiatric disorders. For instance, by the year 2022, the transition probability from the healthy state to psychiatric disorders was 25.3% in the group with ACEs compared to 19.0% in the group with ACEs, representing a 33% increased risk. Univariate Markov models also demonstrated that individuals with ACEs had 19.0%, 25.3% and 85.9% higher risks of transition from No PC & No MS to PC, MS, and to psychiatric disorder, respectively; and 13.8% higher risks of transition from PC to PC & MS. A previous study using multi-state model also found that higher ACE scores (≥ 4) were associated with an increased probability of forward transitions toward worsening physical health states, such as from robust to pre-frail (HR = 1.37, 95% CI: 1.21–1.54) and from pre-frail to frail (HR = 1.39, 95% CI: 1.18–1.63), as well as a decreased probability of reverting to healthier states (pre-frail to robust, HR = 0.64, 95% CI: 0.55–0.76)^42^. Our findings hence extended previous research by dynamically characterizing how ACEs could influence long-term mental health trajectories in older adults, emphasizing the enduring effects of early life stressors on psychiatric morbidity decades later.

Several potential mechanisms may explain the enduring impact of ACEs on late-life mental health trajectories. First, the association between ACEs and poor adult health is likely mediated by psychosocial factors, including having increased exposure to stressful life events, higher perceived stress, negative emotionality, and unhealthy behaviors in adulthood^43^,. Second, chronic stress related to ACEs may induce multisystem dysregulation, including neuroendocrine and inflammatory processes, which cumulatively impair physical and mental health^44^. Third, neuroimaging studies indicates that alterations related to ACEs in brain regions critical for emotional regulation and cognitive control, such as the prefrontal cortex and hippocampus, may mediate increased vulnerability to psychiatric disorders, particularly in individuals experiencing cognitive decline^13, 14^. These psychosocial and biological processes can cumulatively generate sustained risk factors, including chronic inflammation, maladaptive coping strategies, and health-risk behaviors, that may delay recovery from mental health challenges or accelerate transitions to psychiatric disorders.

Subgroup analyses revealed a strong association between ACEs and the incidence of psychiatric disorders among individuals aged 50–64 years and those with higher educational attainment. This aligns with the results of our multi-state model, showing that individuals aged 50–64 years with ACEs had greater risks of transitioning from all health states to psychiatric disorders, while those with a higher education level and ACEs had greater risks of transitioning from MS or PC & MS to psychiatric disorders. Individuals aged 50–64 years often experience greater work-related stress, family obligations, and caregiving responsibilities, compared to those aged 65 and older. Such stressors, combined with psychosocial vulnerabilities resulting from ACEs, can contribute to increased emotional distress and risk of psychiatric disorders^43^. Individuals with a higher education level may exhibit greater awareness of mental health symptoms and are more likely to seek help, leading to increased rates of psychiatric disorder diagnosis^45^. Furthermore, despite having socioeconomic advantages, individuals with a higher education level may still experience major psychosocial stressors (e.g., career pressure and social expectations) that can exacerbate the impact of early adversities^46^. Our findings that females with ACEs had increased risks of transitions from all health states to psychiatric disorders is also consistent with the higher prevalence of mental illness observed in females^47^. Biological sensitivities, including hormonal fluctuations and neural differences, combined with gender-specific psychosocial stressors such as caregiving burdens and exposure to gender-based violence, can amplify the impact of early adversities on emotional regulation and psychiatric vulnerability in women^48, 49^. The interaction between ACEs and female-specific factors might contribute to the elevated psychiatric risk across various health trajectories observed in our study.

Furthermore, in the group with ACEs, univariate multi-state analysis identified other factors that significantly influenced the risk of transition to psychiatric disorders. Behavioral factors, such as physical inactivity and insomnia, could further increase psychiatric risk, highlighting the role of lifestyle in modulating outcomes of ACEs. Chronic physical conditions, including lung diseases and arthritis, also elevated the risk of progression profoundly from healthy (No PC & No MS) or physical condition (PC) states to psychiatric disorders, potentially through mechanisms related to systemic inflammation^50^ and functional impairment^51^.

From a clinical perspective, our findings highlight the importance of routine screening for ACEs in older adults to identify those with elevated risk for psychiatric disorders, especially among individuals exposed to trauma, violence and family dysfunction in early life. Behavioral interventions targeting those with risk factors such as younger age, female gender, and high education level as well as modifiable factors such as physical activity and sleep disturbances, may mitigate the development of psychiatric morbidity. Further, effective management of chronic physical conditions like lung diseases and arthritis can be crucial to reduce the transition towards psychiatric disorders. Future research to explore integrated care models addressing both multimorbidity and psychosocial adversities is warranted to improve the mental health outcomes in older populations with ACEs.

The strengths of this study included a large, nationally representative cohort with longitudinal data spanning over two decades, the use of a comprehensive set of time-varying covariates to reduce potential confounding, and the application of multi-state Markov modeling that enabled a nuanced understanding of dynamic progress to the onset of psychiatric disorders. However, some limitations should be noted. First, ACEs were retrospectively self-reported, which might introduce recall bias. However, a prior study has reported on the reasonable validity of adult retrospective reporting of ACEs^52^. Similar to previous research^26, 53^, psychiatric disorders were based on self-reported diagnosis by physicians, which might lead to underreporting. Second, the measurement of ACEs in the HRS included a limited set of adverse events, particularly regarding social problems, which might not fully capture the breadth of childhood adversities. Third, potential confounding factors that might influence health trajectories, such as genetic predisposition, social support or later-life stressors, were not included in the analyses. Fourth, missing data in certain time-varying covariates (i.e., CESD-8, JSS-4, and TICS-m) were imputed using LOCF and NOCF methods, which might introduce bias regarding the constancy of data across time. Fifth, due to limited number of transitions between health states, we were unable to perform multivariate Markov modeling incorporating all variables that were significantly associated with transitions in the univariate analyses, thus limiting the adjustment for confounding factors. Finally, although the cohort is representative of the US older adult population, findings might not be generalizable to those in other cultural or socioeconomic contexts.

In conclusion, this long-term cohort study revealed that ACEs could significantly increase the risk of PDOA as well as accelerate the progression to PDOA, especially among those exposed to past trauma and violence and family dysfunction. The association between ACEs and PDOA were more pronounced among females, participants aged 50–64 years, and those with higher education level or with cognitive decline. Individuals with ACEs showed faster transitions from healthy and intermediate states to having psychiatric disorders, spent less time in healthy state, and had prolonged periods of multimorbidity prior to onset of psychiatric disorder. Demographic, behavioral, and physical health factors (such as age, sex, education level, physical activity, insomnia, lung diseases, and arthritis) might further modify these risks. Our findings highlighted the enduring impact of early life adversity on mental health trajectories in later life, and further underscored the need for integrated, trauma-informed care to mitigate psychiatric morbidity in older populations.

## Supplementary Material

Supplementary Files

This is a list of supplementary files associated with this preprint. Click to download.

- SupplementaryMaterials.docx

## Figures and Tables

**Table 1:** Baseline characteristics of the total sample and by different types of ACEs (N = 8,628)

Variables	Total (N = 8,628)		Types of ACEs								Univariate analyses	
			No adversity (N = 5.672, 65.7%)		Trauma & violence (N = 2,160, 25.0%)		Family dysfunction (N = 608, 7.0%)		Social problems (N = 188, 2.2%)			
	N	%	N	%	N	%	N	%	N	%	*χ* ^2^	*P*
Male	3,692	42.8	2,158	38.0	312	53.6	577	41.6	645	65.3	284.920	**<0.001**
Race											14.143	**0.028**
White/Caucasian	7,331	85.0	4,785	84.4	516	88.7	1,192	86.0	838	84.8		
Black/African American	1,005	11.6	689	12.1	44	7.6	149	10.8	123	12.4		
Other	291	3.4	197	3.5	22	3.8	45	3.2	27	2.7		
Married	6,310	73.1	4,120	72.6	451	77.5	974	70.3	765	77.4	21.382	**<0.001**
High school education or above (≥12 years)	3,727	43.2	2,562	45.2	287	49.3	591	42.6	287	29.0	98.640	**<0.001**
Living with others	6,680	77.4	4,354	76.8	473	81.3	1,049	75.7	804	81.4	17.573	**<0.001**
Alcohol use	2,801	32.5	1,831	32.3	203	34.9	425	30.7	342	34.6	5.770	0.123
Smoking	1,120	13.0	714	12.6	68	11.7	200	14.4	138	14.0	5.070	0.167
Vigorous physical activity	4499	52.1	2929	51.6	347	59.6	697	50.3	526	53.2	16.007	**0.001**
Physical disease												
Diabetes	958	11.1	615	10.8	59	10.1	151	10.9	133	13.5	6.568	0.087
Cancer	848	9.8	563	9.9	57	9.8	147	10.6	81	8.2	3.970	0.265
Heart conditions	1298	15.0	818	14.4	104	17.9	208	15.0	168	17.0	8.325	**0.040**
Stroke	260	3.0	169	3.0	18	3.1	39	2.8	34	3.4	0.843	0.839
Hypertension	3857	44.7	2529	44.6	238	40.9	627	45.2	463	46.9	5.472	0.140
Lung diseases	482	5.6	293	5.2	41	7.0	93	6.7	55	5.6	7.568	0.056
Arthritis	4582	53.1	2979	52.5	327	56.2	739	53.3	537	54.4	3.637	0.304
	Mean	SD	Mean	SD	Mean	SD	Mean	SD	Mean	SD	t/Z	P
Age (years)	64.75	8.01	65.22	8.11	65.55	8.34	62.82	7.55	64.28	7.40	106.660	**<0.001**
Depression (CESD-8 total)	1.14	1.59	1.07	1.54	1.22	1.64	1.31	1.75	1.25	1.63	27.831*	**<0.001**
Sleep (JSS-4 total)	6.71	2.03	6.69	2.02	6.88	2.04	6.69	2.08	6.73	2.00	5.549*	0.136
Cognition (TICS-m)	16.08	4.21	16.24	4.20	16.25	4.15	16.13	4.19	15.00	4.18	73.380*	**<0.001**

Notes: Bolded values indicate *P* < 0.05; ACE: adverse childhood experience; SD: standard deviation. CESD-8: 8-item Center for Epidemiological Studies Depression Scale; JSS-4: 4-item Jenkins Sleep Scale; TICS-m: modified version of the Telephone Interview for Cognitive Status Scale.

* Analyzed using Wilcoxon rank sum test.

**Table 2:** The associations between types of ACEs and the incidence of PDOA (N = 8,628)

Variables	Cases N	Incidence rate, per 1000 person-years	Model 1			Model 2			Model 3		
			HR	95%CI	*P*	HR	95%CI	*P*	HR	95%CI	*P*
ACE type											
No adversity	5,672	8.85	Reference			Reference			Reference		
Trauma & violence	582	11.4	1.372	1.123–1.678	**0.002**	1.384	1.132–1.693	**0.002**	1.279	1.052–1.555	**0.014**
Family dysfunction	1,386	14.1	1.607	1.412–1.830	**<0.001**	1.557	1.366–1.775	**<0.001**	1.358	1.190–1.548	**<0.001**
Social problems	988	8.92	1.152	0.964–1.377	0.119	1.099	0.918–1.317	0.305	0.974	0.813–1.167	0.772
Age (years)			0.994	0.987–1.001	0.091	0.982	0.974–0.989	**<0.001**	0.970	0.962–0.978	**<0.001**
Male			0.637	0.568–0.713	**<0.001**	0.777	0.689–0.876	**<0.001**	0.768	0.680–0.867	**<0.001**
Race											
White/Caucasian			Reference			Reference			Reference		
Black/African American			0.765	0.641–0.914	**0.003**	0.649	0.541–0.778	**<0.001**	0.544	0.452–0.654	**<0.001**
Other			1.095	0.834–1.438	0.515	0.966	0.734–1.270	0.802	0.839	0.646–1.090	0.189
Married						0.672	0.500–0.904	**0.009**	0.787	0.580–1.068	0.125
High school education or above (≥ 12 years)						0.945	0.847–1.054	0.309	1.176	1.049–1.320	**0.006**
Living with others						1.115	0.825–1.507	0.478	1.154	0.848–1.571	0.362
Alcohol use						0.624	0.549–0.708	**<0.001**	0.703	0.619–0.799	**<0.001**
Smoking						1.060	0.883–1.274	0.531	0.949	0.788–1.143	0.578
Vigorous physical activity						0.634	0.563–0.714	**<0.001**	0.815	0.721–0.921	**<0.001**
Physical disease											
Diabetes									0.947	0.836–1.072	0.387
Cancer									0.882	0.769–1.013	0.075
Heart conditions									1.127	1.004–1.266	**0.043**
Stroke									1.443	1.226–1.698	**<0.001**
Hypertension									1.102	0.977–1.243	0.116
Lung diseases									1.253	1.081–1.452	**0.003**
Arthritis									1.294	1.136–1.474	**<0.001**
Depression score (CESD-8)									1.317	1.285–1.349	**<0.001**
Sleep score (JSS-4)									1.067	1.039–1.096	**<0.001**
Cognition score (TICS-m)									0.961	0.948–0.974	**<0.001**

Notes: Bolded values indicate *P* < 0.05. ACE: adverse childhood experience; PDOA: psychiatric disorders in older adults; HR: Hazard ratio; CI: Confidence interval; CESD-8: 8-item Center for Epidemiological Studies Depression Scale; JSS-4: 4-item Jenkins Sleep Scale; TICS-m: modified version of the Telephone Interview for Cognitive Status Scale.

Model 1 was adjusted for age, sex and race/ethnicity.

Model 2 was adjusted for age, sex, race/ethnicity, marital status, living with others, educational level, vigorous activities, smoking, and alcohol use.

Model 3 was adjusted for the same covariates as model 2 plus history of major physical diseases (diabetes, cancer, heart conditions, stroke, hypertension, lung diseases, and arthritis), total CESD-8 score, total JSS-4 score and total TICS-m score.

## Data Availability

The data that support the findings of this study are available from The University of Michigan Health and Retirement Study Platform at https://hrsdata.isr.umich.edu/data-products/public-survey-data
